# V-akt murine thymoma viral oncogene homolog 3 (AKT3) contributes to poor disease outcome in humans and mice with pneumococcal meningitis

**DOI:** 10.1186/s40478-016-0320-9

**Published:** 2016-05-18

**Authors:** Mercedes Valls Serón, Bart Ferwerda, JooYeon Engelen-Lee, Madelijn Geldhoff, Valery Jaspers, Aeilko H. Zwinderman, Michael W. Tanck, Frank Baas, Arie van der Ende, Matthijs C. Brouwer, Diederik van de Beek

**Affiliations:** Department of Neurology, Center of Infection and Immunity Amsterdam (CINIMA), Academic Medical Center, University of Amsterdam, P.O. Box 22660, 1100DD Amsterdam, The Netherlands; Department of Clinical Epidemiology, Biostatistics, and Bioinformatics, Academic Medical Center, University of Amsterdam, Amsterdam, The Netherlands; Department of Genome Analysis, Academic Medical Center, Amsterdam, The Netherlands; Department of Medical Microbiology, Center of Infection and Immunity Amsterdam (CINIMA), Academic Medical Center, Amsterdam, The Netherlands; The Netherlands Reference Laboratory for Bacterial Meningitis, Center of Infection and Immunity Amsterdam (CINIMA), Academic Medical Center, Amsterdam, The Netherlands

**Keywords:** Bacterial meningitis, *Streptococcus pneumoniae*, Prospective nationwide genetic association study, AKT3

## Abstract

Pneumococcal meningitis is the most common and severe form of bacterial meningitis. Fatality rates are substantial, and long-term sequelae develop in about half of survivors. Here, we have performed a prospective nationwide genetic association study using the Human Exome BeadChip and identified gene variants in encoding dynactin 4 (*DCTN4*), retinoic acid early transcript 1E (*RAET1E*), and V-akt murine thymoma viral oncogene homolog 3 (*AKT3*) to be associated with unfavourable outcome in patients with pneumococcal meningitis. No clinical replication cohort is available, so we validated the role of one of these targets, *AKT3*, in a pneumococcal meningitis mouse model. *Akt3* deficient mice had worse survival and increased histopathology scores for parenchymal damage (infiltration) and vascular infiltration (large meningeal artery inflammation) but similar bacterial loads, cytokine responses, compared to wild-type mice. We found no differences in cerebrospinal fluid cytokine levels between patients with risk or non-risk alleles. Patients with the risk genotype (rs10157763, AA) presented with low scores on the Glasgow Coma Scale and high rate of epileptic seizures*.* Thus, our results show that AKT3 influences outcome of pneumococcal meningitis.

**Electronic supplementary material:**

The online version of this article (doi:10.1186/s40478-016-0320-9) contains supplementary material, which is available to authorized users.

## Introduction

Bacterial meningitis is a severe infection of the central nervous system, which is caused by *Streptococcus pneumoniae* in 70 % of cases [1]. Patients with pneumococcal meningitis are at high risk for complications, such as epileptic seizures and cerebral infarction, resulting in a high mortality rates, and neurologic sequelae, including hearing loss, focal neurological deficit, and cognitive impairment, in almost half of survivors [2–7]. Following the introduction of adjunctive dexamethasone treatment the mortality rate of pneumococcal meningitis has decreased from 30 to 20 % [8–10], but new treatments are urgently needed [11–13]. Interindividual differences in severity of disease and outcome may be determined by host genetic variation [14, 15]. So far, genetic association studies in pneumococcal meningitis have used a candidate gene approach in which variations in genes of interest were studied [15–17], focusing on genes in the immune system, for example the toll-like receptor signalling cascade and the complement system [14, 15].

Whole-genome association studies, using an unbiased approach, may identify new candidate genes and pathophysiological mechanisms leading to an unfavourable outcome in pneumococcal meningitis [17]. Such studies should be performed prospectively, using a clear definition of cases with microbiological confirmation, and validated outcome scales [18]. In 2006, we started a nationwide prospective cohort study to identify and characterize host genetic traits and bacterial genetic factors controlling occurrence and outcome of bacterial meningitis (MeninGene) [9, 18]. Here, we report our genome wide association study on the host side, analysing the associations between human genome variants and functional outcome in patients with pneumococcal meningitis using the Human Exome BeadChip. We subsequently validated the role of one of the top hits, *AKT3*, in a pneumococcal meningitis mouse model. Finally, we studied whether patients with the associated *AKT3* rs10157763 risk allele had specific clinical characteristics or cerebrospinal fluid (CSF) inflammatory marker profiles.

## Materials and methods

### Dutch bacterial meningitis cohort

In a nationwide prospective cohort study (MeninGene) we included patients with community-acquired bacterial meningitis with an age of 16 years or older with positive CSF cultures who were identified by The Netherlands Reference Laboratory for Bacterial Meningitis (NRLBM) from March 2006 to October 2011 [14]. The NRLBM receives bacterial isolates from approximately 85 % of bacterial meningitis patients in the Netherlands and provided the names of the hospitals where patients with bacterial meningitis had been admitted in the previous 2–6 days. Physicians of the hospitals were contacted after which the treating physician obtained informed consent from the patient or their legal representative. Online case record forms were collected through a secured website and included data on symptoms during the hospitalized period, treatment, complications and outcome. Patients with hospital-acquired bacterial meningitis and negative CSF cultures were excluded. Outcome was graded at discharge according to the Glasgow Outcome Scale (GOS), a well validated instrument with good inter-observer agreement [19]. On this five-point scale a score of 1 indicates death, 2 a vegetative state, 3 severe disability, 4 moderate disability, and a score of 5 mild or no disability.

Blood for DNA extraction was collected in sodium/EDTA tubes and DNA-isolation was performed with the Gentra Puregene isolation kit (Qiagen) according to manufacturer’s protocol thereafter the yield and quality of the extractions were determined to ensure appropriate conditions for genotyping.

### Genotyping and quality control

Patients were genotyped on the Illumina HumanExome BeadChip v1.1 consisting of >24,0000 markers, with approximately 75 % of these markers having a minor allele frequency (MAF) < 0.05. The genotyping was carried out in collaboration with the Human Genome Facility and the department of Epidemiology, Erasmus MC, the Netherlands as part of the Netherlands ExomeChip Project. After genotyping we performed genotype-calling on all our MeninGene samples (*N* = 1322) to improve calling accuracy and all non-pneumococcal meningitis patients were removed after quality control (QC). All autosomal SNPs were called using GenomeStudio software, from Illumina, with the GenTrain2.0 cluster algorithm using the protocol as described before [20].

Called data was exported in PLINK format for final sample and SNP QC [21]. All samples with > 5 % missing genotypes were removed. High quality, independent SNPs with less than 1 % missing-genotypes, non-significant differences between cases and controls with respect to rate of missing genotypes, MAF >5 % and Hardy-Weinberg equilibrium (HWE) *p*-value > 1 × 10^−3^ were used to determine relatedness between patients, heterozygosity and ancestry. Individuals with second degree, or higher degree relatedness, heterozygosity above or below three standard deviations and non-European decent were left out. Finally QC was performed by removing all SNPs with >5 % missing genotypes and HWE *p*-value < 1 × 10^−6^.

### Mice

8- to 12-week-old male C57BL/6 N mice were obtained from Charles River, whereas *Akt3*^-/-^ mice (backcrossed at least 10 times to a C57BL/6 N background), generated as described elsewhere [22] were bred and maintained at the animal facility at the Academic Medical Center of Amsterdam. The mice were kept to a controlled 12 h light/dark cycle and food and water were provided *ad libitum.*

### Experimental pneumococcal meningitis model

Pneumococcal meningitis was induced by intracisternal inoculation with *S. pneumoniae* (serotype 3, American Type Culture Collection #6303) as described previously [18, 23–25]. In brief, wild-type and *Akt3*^-/-^ mice (*n* = 12 per group) were randomly and blind inoculated with 1 μl bacterial suspension containing 1 × 10^4^ CFU *S. pneumoniae* into the cisterna magna under Isofluran (Baxter) anaesthesia. Six mice per group inoculated with sterile saline (Baxter) were used as controls*.* Cultures were adjusted such that each final 1 μl inoculum contained 1 × 10^4^ CFU. Immediately after intracisternal inoculation, mice were assessed for neurologic damage as a result of the puncture. No mice suffered from neurological damage as a result of puncture and had to be excluded. Mice were observed during 48 h post infection and clinical signs of meningitis were blindly scored every four hours as previously described [23]. In brief, the maximum score was determined by the estimated contribution of the variable to overall health of the mouse: weight loss (0–4 points), activity (0–4 points), time to return to upright position (0–6 points), state of skin/fur (0–3 points), posture (0–2 points), eye discharge or protrusion (0–4 points), respiration rate (0–4 points), irregular/labored breathing (0–4 points), epilepsy, limb paresis or ataxia (0–10 points). Animals reaching endpoint criteria were humanely killed. In another set of experiments, at 6, 24 and 30 h post infection, 12 mice/group were anesthetized by intraperitoneal injection of 190 mg/kg ketamine (Eurovet Animal Health) and 0.3 mg/kg dexmedetomidine (Pfizer Animal Health) followed by cardiac puncture for blood collection and perfusion of organs with sterile isotonic saline via the left ventricle. CSF was collected by puncture of the cisterna magna. Brain, lung and spleen were harvested, placed on ice, processed and stored as described before [23]. EDTA blood was centrifuged at 2000 × g for 15 min. Plasma was stored at −80 °C for further analysis.

### Real-time PCR

Total RNA was extracted from murine brain homogenates using TriPure reagent (Sigma-Aldrich). The purity of the RNA was assessed by the ratio of absorbance at 260 and 280 nm. RNA purity was within a range of 2.0–2.1. RNA was stored in aliquots at −80 °C until used for reverse transcription. For complementary DNA (cDNA) synthesis, RNA was treated with RQ1 RNase-free DNase (Promega) and reverse transcribed with SuperScript II Reverse Transcriptase and random hexamers (Life Technologies). The real-time polymerase chain reaction (RT-PCR) measurement of individual cDNAs was performed on a Bio-Rad MyiQ Single-Color RT-PCR Detection System using the Bio-Rad iQ SYBR Green Supermix (Bio-Rad Laboratories). The Non-POU-domain containing octamer binding protein (*nono*, housekeeping gene) primers were described previously [26]. *Akt3* cDNA was amplified with a forward 5′-CATCTGAAACAGACACCCGATA-3′ and a reverse 5′-GTCCGCTTGCAGAGTAGGAG-3′ primer for mouse *akt3. Dctn4* cDNA was amplified with a forward 5′- CACCCATGTGACTGTGTTGG and a reverse 5′-TGAAGATGCCCACTTTGTTG primer for a mouse *dctn4. Raet1E* was amplified with a forward 5′-GGATACACCAACGGGCTG and a reverse 5′-GTCACACCCAGGGAAGGTC primer for a mouse *raet1e* (all Applied Biosystems). A negative control without the Reverse Transcriptase was also used. The expression data were normalized to the *nono* reference gene. Data were analyzed using the Bio-Rad MyiQ Optical system Software version 1.0.

### Murine histopathology and immunohistochemistry

Histopathology was performed on the right cerebral hemisphere fixed in 4 % paraformaldehyde and paraffin embedded. Coronal 5 μm sections of the right hemisphere were cut for subsequent staining with hematoxylin and eosin (HE) according to standard procedures. HE staining were performed to visualize meningeal infiltration, parenchymal damage, vascular inflammation, thrombosis and ventriculitis on a three point scale (Additional file 1). Sections of mice 24 h after induction of pneumococcal meningitis (*n* = 12 per group) were scored. Tissue sections were stained for Caspase-3 using immunohistochemical procedures in order to assess hippocampal apoptosis, as described previously [24].

### Determination of cytokines and chemokines

The murine cytokines interleukin (IL)-1β, IL-6, IL-10 and tumour-necrosis factor-α (TNF-α) and the chemokines keratinocyte chemoattractant (KC) and macrophage inflammatory protein (MIP)-2 were determined in plasma and brain homogenates by Luminex technology using a mouse Bioplex kit (Bio-Rad Laboratories) as described before [25]. The human cytokines IL-1β, IL-6, IL-10 and TNF-α were determined in CSF collected from the diagnostic lumbar puncture. CSF samples were centrifuged and supernatant was aliquoted and stored at −80 °C until analysis. All analytes were measured with Luminex**®** xMAP**®** technology using Milliplex**®** map multiplex assay’s (Millipore Billerica). CSF was diluted 1:50 in assay buffer provided with the Multiplex assay and inflammatory mediators were measured according to the manufacturer’s instructions. CSF was available in 271 of the 689 (39 %) of pneumococcal meningitis patients.

### Statistics

All patients with pneumococcal meningitis were included in the genetic association analysis for meningitis disease severity/outcome. Severity/outcome was determined by grouping patients on their GOS score where score 1 until 4 together was labelled unfavourable and score 5 as favourable outcome. All calculations have been conducted using PLINK 1.9 [27]. Differences between favourable and unfavourable outcome were calculated using the Fisher’s exact test on allelic association. For the genetic association study, based on 321 patients with a favourable outcome and 152 with unfavourable outcome, for 240,000 variants and a MAF of 0.25, we had 80 % power to detect variants with a OR of 3.4 or higher after Bonferroni correction. Cytokine level differences between genotypes were compared using a Mann-Whitney *U* test.

We used a multivariable logistic regression analysis to calculate OR and 95 % CI to assess the strength of the association among potential risk factors (including identified polymorphisms) and outcome. Murine gene expression, bacterial load and inflammatory response data were compared using Mann-Whitney U tests. Survival curves were compared using a log rank test and clinical scores were compared using a linear mixed model assuming an exponential and group specific time effect. For all analyses, SPSS (version 22) was used; differences were considered statistically significant at *P* < 0.05.

## Results

### Nationwide prospective cohort study

In a prospective nationwide study we included 1005 episodes of culture-proven community-acquired bacterial meningitis in 993 patients. The distribution of causative bacteria was *S. pneumoniae* in 720 (72 %), *Neisseria meningitidis* in 105 (11 %), and other bacteria in 180 (18 %) episodes. DNA samples were obtained from 472 pneumococcal meningitis patients (66 %) of whom 430 were from Caucasian population (91 %). The mean age of pneumococcal meningitis patients for whom DNA was available was 58 years and 221 (47 %) were male (Table 1). Predisposing conditions, most commonly otitis or sinusitis (218 episodes, 46 %) or an immunocompromised state (124 episodes, 26 %) were present in 322 episodes (68 %). Upon presentation epileptic seizures occurred in 33 patients (7 %). During clinical course, new focal neurologic deficits developed in 24 % of patients. Outcome was prospectively evaluated using a validated outcome scale [19], the GOS, a score of 1 to 4 was defined as unfavourable outcome, a score 5 as favourable outcome), in all 472 patients with pneumococcal meningitis with DNA available: 151 (32 %) patients had an unfavourable outcome and 37 (8 %) died.

**Table 1:** Clinical characteristics of 472 patients with community-acquired pneumococcal meningitis^a^

Characteristic		Characteristic	
Age – yr	58 ± 15	Indexes of CSF inflammation^c^	
Male sex – no (%)	221 (47 %)	Opening pressure (mm H_2_O)	42 ± 11
Duration of symptoms <24 h	225/461 (49 %)	White blood cell count (/mm^3^)	2417 ± 5648
Pretreatment with antibiotics	58/459 (13 %)	<1.000/mm^3^	130/454 (29 %)
Predisposing conditions	322/472 (68 %)	Protein – g/L	4.7 ± 3.2
Otitis or sinusitis	218/471 (46 %)	CSF: blood glucose ratio	0.13 ± 0.18
Immunocompromised	124/472 (26 %)	Positive blood cultures	337/420 (80 %)
Symptoms and signs on presentation^b^		Complications	
Headache	363/427 (85 %)	Cardiorespiratory failure	170/466 (36 %)
Neck stiffness	356/454 (78 %)	Focal neurologic deficits	109/455 (24 %)
Epileptic seizures	33/454 (7 %)	Epileptic seizures	69/460 (15 %)
Systolic blood pressure – mmHg	149 ± 29	Score on Glasgow Outcome Scale	
Heart rate – beats/min	100 ± 21	1 – death	37 (8 %)
Body temperature – °C	38.8 ± 1.3	2 – vegetative state	1 (0.2 %)
Score on Glasgow Coma Scale^c^	11 ± 3	3 – severe disability	23 (5 %)
<8 indicating coma	63/472/87 %)	4 – moderate disability	90 (19 %)
Focal neurologic deficits	109/455 (25 %)	5 – good recovery	321 (68 %)

^a^ Data are number/number evaluated (percentage), continuous data are mean ± SD. ^b^Systolic blood pressure was evaluated in 469 patients, heart rate in 466, temperature in 471. ^c^CSF opening pressure was evaluated in 165 patients, CSF WBC count in 454 patients, CSF protein in 452, CSF blood: glucose ratio in 452

### Genetic association study

A total of 472 patients with pneumococcal meningitis were genotyped on the Illumina Human Exome BeadChip. Of the top five candidate single nucleotide polymorphisms (SNPs) associated with unfavourable outcome (Table 2) two pairs of candidate SNPs were in linkage disequilibrium (LD), the rs11954652 and rs6869603 within the *DCTN4* gene (odds ratio [OR] 5.6 for unfavourable outcome, 95 % confidence interval [CI] 2.4–12.9, *p* = 2.4× 10^−5^). Rs11954652 is a missense variant and rs6869603 is a variant within a regulatory element before the *DCTN4* gene. SNPs rs3798763 and rs6925151 are also in LD and lay within the *RAET1E* gene (OR 1.9 for unfavourable outcome, 95 % CI 1.4–2.6, *p* =9.4× 10^−5^). Rs3798763 is an intron variant and rs6925151 is missense variant. Rs10157763 is located within the intron of the *AKT3* gene (OR 1.88 for unfavourable outcome, 95 % CI 1.4–2.6, *p* = 9.9× 10^−5^). The missense variations within *DCTN4* and *RAET1E* had no predicted impact of amino acid substitution on structure or function of the gene using PolyPhen-2 [28]. The potential effect of other three variations on the protein structure or function was unclear.

**Table 2:** Novel SNPs associated with unfavorable outcome in patients with pneumococcal meningitis

Gene	Chr	Variation type	dbSNIPID	BP	Alleles		Allele frequency		*P*	BONF_P	OR (+/− 95 CI)
					Effect^d^	Other^e^	UF	F			
*DCTN4* ^*a*^	5	Upstream gene variant	rs6869603^b^	150142849	G	A	0.07	0.01	2.4 × 10^−05^	1	5.6 (2.4–12.9)
*DCTN4*	5	F349L, F342L, F285L	rs11954652^b^	150097883	G	C	0.07	0.01	2.4 × 10^−05^	1	5.6 (2.4–12.9)
*RAET1E*	6	E89E, E53E	rs3798763^c^	150211100	G	A	0.37	0.23	9.4 × 10^−05^	1	1.9 (1.4–2.6)
*RAET1E*	6	R128H, R92H	rs6925151^c^	150210723	G	A	0.37	0.23	9.4 × 10^−05^	1	1.9 (1.4–2.6)
*AKT3*	1	Silent	rs10157763	243995041	A	G	0.40	0.26	9.9 × 10^−05^	1	1.9 (1.4–2.6)

BP denotes base pair, UF unfavourable outcome, F favourable outcome

^a^ Variation within a regulatory area of this gene

^b^,^c^ Markers in linkage disequilibrium (LD)

^d^ Risk allele associated with unfavorable outcome

^e^ Allele without risk

### Role of *akt3* in the pneumococcal meningitis mouse model

**Fig. 1:**
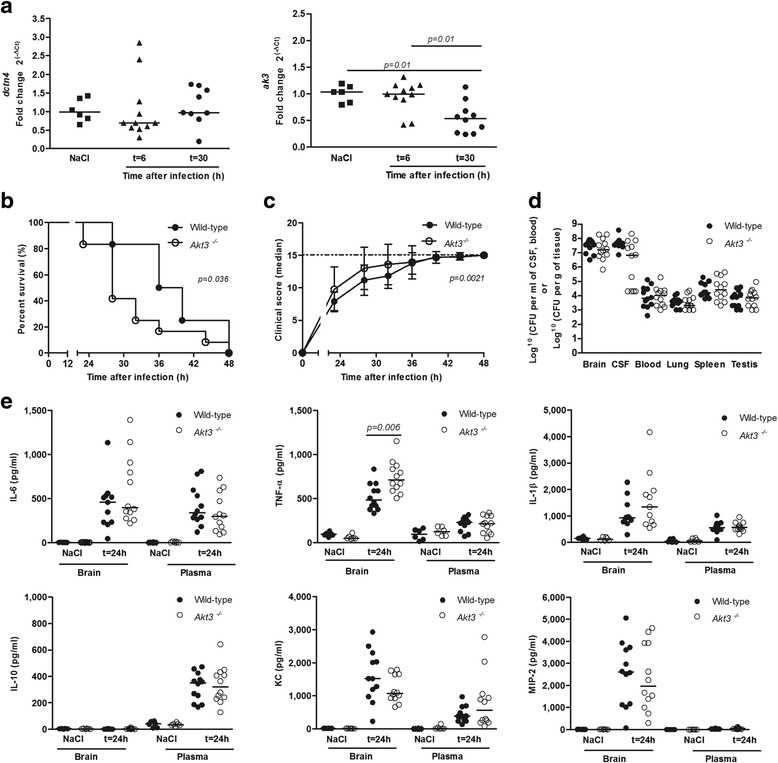
*dctn4* and *akt3* mRNA expression levels and role of *Akt3* in experimental pneumococcal meningitis. **a** Wild-type mice were infected with 1 × 10^4^ colony-forming units (CFU) of *S. pneumoniae* serotype 3, strain 6303 via intracisternal inoculation. mRNA levels were assessed after 6 and 30 h in brain tissue (*n* = 6 control mice and 12 infected mice). **b** Kaplan-Meier survival of mice following intracisternal injection with 1 × 10^4^ CFU of *S. pneumoniae* serotype 3, strain 6303. Survival was monitored two to four times a day over a 48 h period and data analysed using Log-rank test. **c** Clinical scores for each infected mouse group used in the survival study. Scores were compared using a nonlinear mixed effects model, assuming exponential growth (y = β_0_ + e^(β1x)^). **d** Bacterial counts in organs and plasma during *S. pneumoniae* meningitis. **e** Selected cytokine/chemokine levels in brain homogenates of infected mice. Each circle represents a single mouse and the bar indicates the group median expression. Significance was determined using the non-parametric test Mann-Whitney U

**Fig. 2:**
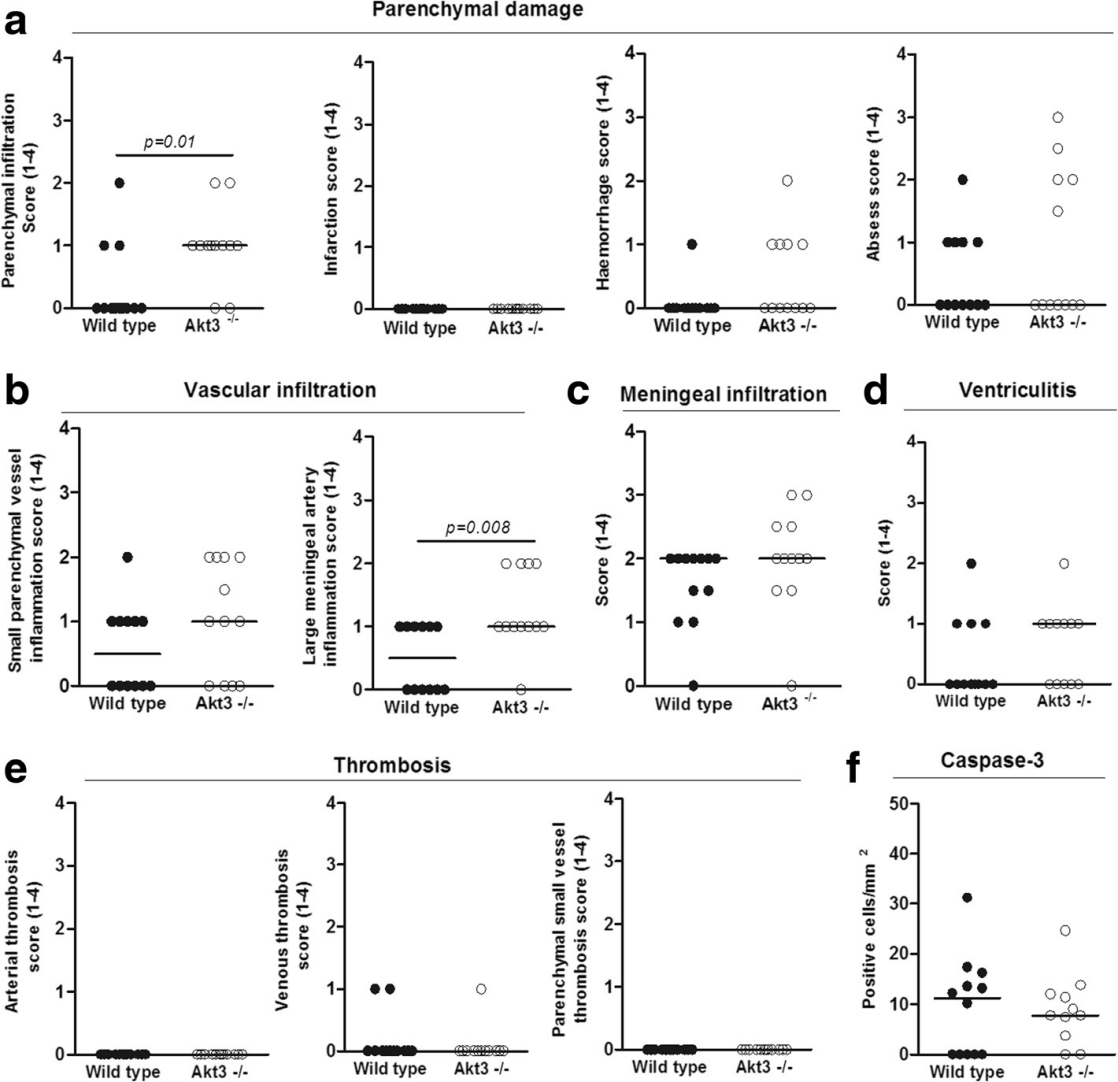
Histopathology scores in *S. pneumoniae*-infected mouse brains. Histopathological changes in mice 24 h after intracisternal injection with 1 × 10^4^ CFU of *S. pneumoniae* serotype 3, strain 6303. Data are represented as median. Statistical analysis was performed using Mann-Whitney *U* test. **a** Parenchymal damage, **b** vascular infiltration, **c** meningeal infiltration, **d** ventriculitis, **e** thrombosis, **f** caspase-3 positive cells

**Fig. 3:**
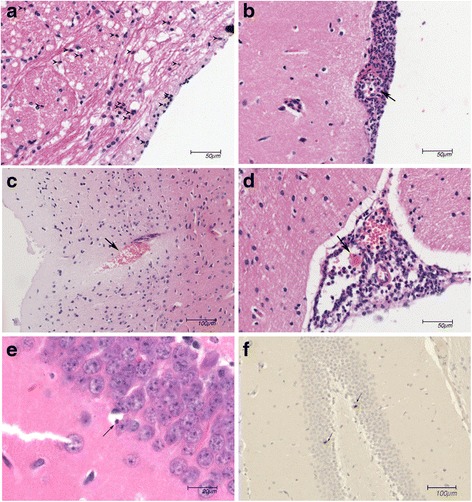
Histopathology during experimental *S. pneumoniae* meningitis. **a**-**e** Representative haematoxylin and eosin and **f** caspase-3- stained sections of a wild-type brain infected with 1 × 10^4^ colony-forming units (CFU) of *S. pneumoniae* serotype 3, strain 6303 via intracisternal inoculation (24 h post inoculation). **a** Red asterisks show parenchymal infiltration of neutrophils. Arrows indicate **b** inflammation of and destruction of meningeal vessel, **c** parenchymal bleeding, **d** thrombosis in meningeal vessel, **e** apoptotic cell with pyknotic nucleus in the dentate gyrus of hippocampus and **f** caspase-3 positive cells in the dentate gyrus of hippocampus

To explore the role of *AKT3, DCTN4*, and *RAET1E* during pneumococcal meningitis we determined their brain expression levels in our mouse model [23]. Mice infected intracisternally with 10^4^ CFUs *S. pneumoniae* ATCC 6303, had low *akt3* mRNA expression in uninfected brain, which decreased 30 h post-infection; *dctn4* brain transcript levels of mice showed no increase after infection (Fig. 1a). No *raet1e* product was detected. Subsequently, we evaluated the functional role of *Akt3* in pneumococcal meningitis in our mouse model using knock-out mice. In a randomized investigator-blinded study, *Akt3*^*-/-*^ mice died faster than wild-type mice when incisternally infected with 10^4^ CFUs of *S. pneumoniae* ATCC 6303 (*p* = 0.036; Fig. 1b). Disease severity scores were also higher in *Akt3*^*-/-*^ mice than wild-type mice (p = 0.002; Fig. 1c). The higher mortality was not caused by differences in bacterial loads or inflammatory response in *Akt3*^*-/-*^ mice as compared with wild-type mice. At 24 h post infection, we observed no difference in bacterial loads in CSF, brain, blood, lung, spleen and testis (Fig. 1d). Levels of TNF-α in brain homogenate levels were higher in *Akt3*^*-/-*^ mice (*p* = 0.006), but IL-1β, IL-6, IL-10, KC and MIP-2 levels were similar between *Akt3*^*-/-*^ and wild-type mice (Fig. 1e). Histopathological examination showed higher histopathology scores for parenchymal damage (infiltration) and vascular infiltration (large meningeal artery inflammation) in *Akt3*^*-/-*^ mice as compared to wild-type mice; there were no differences in parenchymal damage (infarction, haemorrhage, abscess), vascular infiltration (small parenchymal vessel inflammation) meningeal inflammation, ventriculitis, thrombosis, or hippocampal apoptosis between *Akt3*^*-/-*^ and wild-type mice (Figs. 2 and 3).

### *AKT3* rs10157763 variant and risk factors for unfavourable outcome

**Fig. 4:**
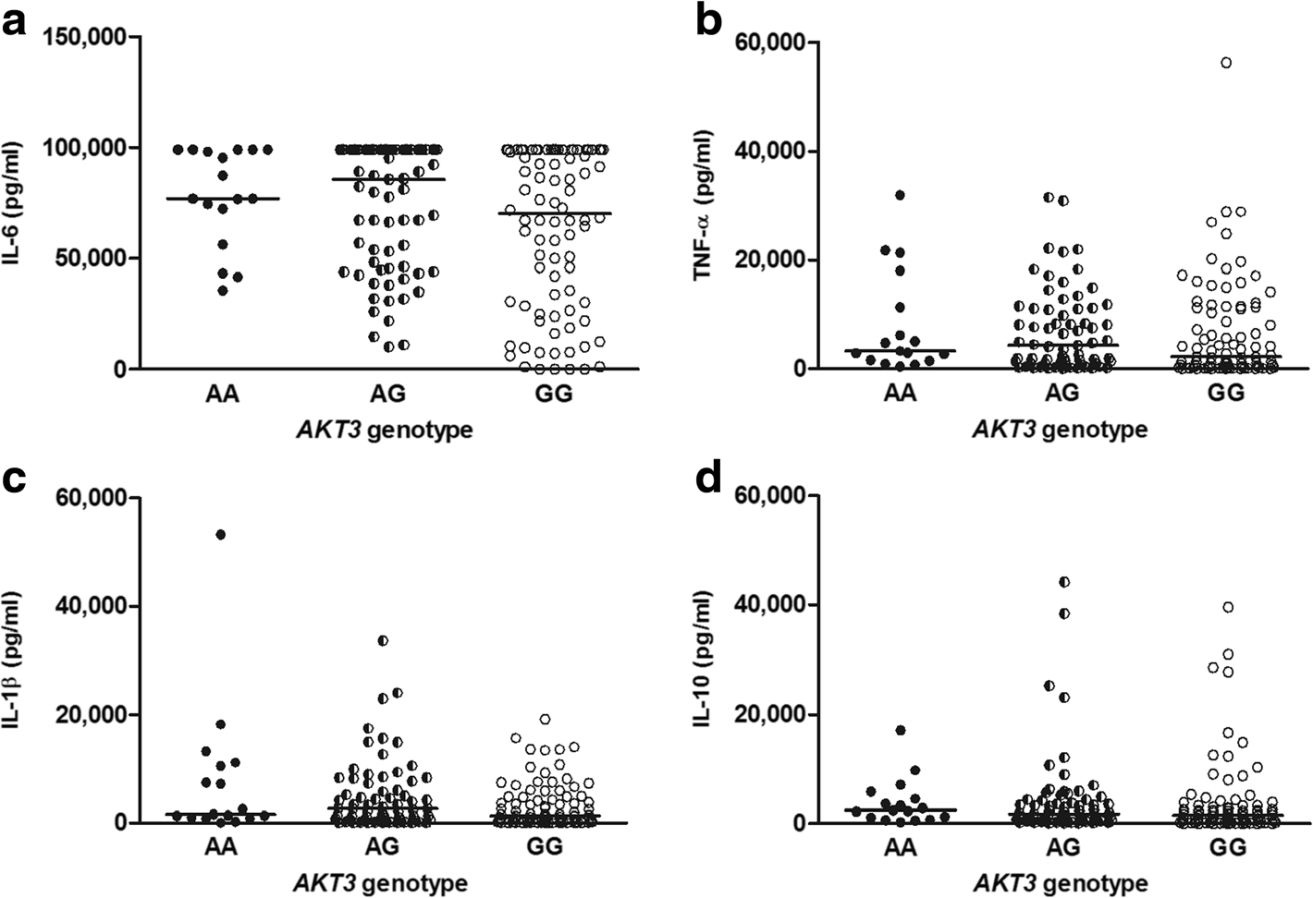
CSF protein cytokine levels between patients with risk or non-risk alleles. **a**-**d** The cytokines IL-6, TNF-α, IL-1β and IL-10 were determined in pneumococcal meningitis patient CSF samples collected from the diagnostic lumbar puncture. Data are represented as median of individual patients grouped on their AKT3 rs10157763 genotypes AA, AG and GG. Statistical analysis was performed using Mann-Whitney *U* test

Having validated the role of *Akt3* in pneumococcal meningitis, we explored the associations between this polymorphism (rs10157763) and clinical features (Table 3). Consistent with our animal data, we found no differences in CSF protein cytokine levels of IL-1β, IL-6 and IL-10 between patients with risk or non-risk alleles at the diagnostic lumbar puncture (Fig. 4). In contrast to the mouse experiments, no differences in CSF TNF-α concentration was observed between genotypes. Patients with the risk genotype (AA: 9.9 %) had an increased risk for seizures (OR 2.94, 95 % CI 1.11–7.80), less frequently exhibited fever (OR 0.43, 95 % CI 0.21–0.84), and had lower scores on the GCS score on admission (median 9 vs 11, *p* = 0.006) as compared with the non-risk genotypes (GG and AG: 40.6 and 49.5 % respectively). In a multivariable regression analysis, including previously identified risk factors for unfavourable outcome (age, CSF white blood cell count < 1000/mm^3^, score on the GC, blood thrombocyte count, immunocompromise, otitis media, and/or sinusitis) [2, 9, 29], the predictive effect of rs10157763 remained robust (OR 3.6, 95 % CI 1.66–7.87; *p* = 0.001).

**Table 3:** Clinical characteristics and outcome per genotype in 396 white patients with community-acquired pneumococcal meningitis^a^

Characteristic	GG (*n* = 195)	AG (*n* = 162)	AA (*n* = 39)	*p*-value recessive model
Age	58 ± 14	60 ± 13	56 ± 17	
Gender	86 (44 %)	79 (49 %)	17 (44 %)	0.63
Glasgow Coma Scale Score	11 ± 3	11 ± 3	9 ± 3	0.006
Seizures on admission	12/188 (7 %)	10/158 (6 %)	6/36 (17 %)	0.024
Fever	168 (86 %)	123 (76 %)	26 (67 %)	0.019
Focal neurologic abnormalities	43 (22 %)	58 (36 %)	14 (36 %)	0.28
Leukocytes <1000	50 (26 %)	52 (32 %)	13 (36 %)	0.36
Respiratory failure	39/191 (21 %)	44/157 (29 %)	13 (33 %)	0.29
Mechanical ventilation	56/186 (30 %)	64/158 (41 %)	18/38 (47 %)	0.19
Seizures during admission	25/192 (14 %)	27/154 (18 %)	5/39 (13 %)	0.76
Focal neurologic deficit during admission	44/188 (23 %)	38/155 (24 %)	15/37 (41 %)	0.02
Unfavourable outcome	57/195 (29 %)	52/162 (32 %)	22/38 (58 %)	0.001
Death	12/195 (6 %)	13/162 (8 %)	4/38 (11 %)	0.43

## Discussion

In the first genome wide association study on outcome in pneumococcal meningitis gene variants in *AKT3, DCTN4* and *RAET1E* were associated with unfavourable outcome in adults with pneumococcal meningitis. As no clinical cohort is available for validation [18], we explored our findings in our pneumococcal meningitis mouse model. The identified downregulation and increased mortality during experimental meningitis confirmed a role of Akt3 during pneumococcal meningitis, although the exact mechanism remains unclear.

In pneumococcal meningitis, brain damage and unfavourable outcome result from massive neutrophil recruitment and inflammation of the brain, reflected by CSF pleocytosis, seizures, and cerebrovascular complications [30, 31]. Patients with the *AKT3* risk allele had a higher level of disease severity, consisting of lower level of consciousness, and a higher risk for epileptic seizures. AKT3 is a critical signalling protein in the Phosphoinositide 3-kinase pathway involved in diverse cellular process such as cell survival, growth, proliferation, metabolism and migration [32]. Inhibition of AKT has been shown to promote apoptosis in many cell types [33, 34].

AKT has been shown to be essential to induce neutrophil extracellular traps, which are pro-inflammatory and important to kill bacteria [35]. In a model of myelin-oligodendrocyte glycoprotein–induced experimental autoimmune encephalomyelitis (EAE), *Akt3*^-/-^ mice were more severely affected than wild-type mice [36]. Experiments using bone marrow chimeras demonstrated that *Akt3*^-/-^ mice receiving *Akt3*-deficient bone marrow cells had elevated clinical scores relative to control wild-type mice reconstituted with wild-type cells, indicating that altered function of both central nervous system (CNS) cells and bone marrow-derived immune cells contributed to the phenotype [36]. These experiments showed that *Akt3*^-/-^ mice have increased inflammatory cytokine expression in spinal cord during acute EAE [36]. In our experiments, *Akt3*^-/-^ mice had similar bacterial outgrowth and mild host inflammatory response as compared to wild-type mice. For neuropathology, we only evaluated a relatively early time-point (24 h) without antibiotic treatment in which gross pathology usually do not present yet. As knockout mice started to die after 24 h however, we were unable to postpone this time point without introducing selection bias in our results. Nevertheless, *Akt3*^-/-^ mice showed increased parenchymal infiltration and artery inflammation as compared to wild-type mice, suggesting an important role of AKT3 in brain integrity and inflammation. In line, a plausible mechanism in which AKT3 is involved in meningitis is by influencing the inflammatory response through influencing apoptosis of inflammatory cells.

An alternative explanation for the poor prognosis associated with AKT3 could be a decreased seizure threshold. Enhanced activation of the mammalian target of rapamycin (mTOR)-signalling cascade has been identified in focal malformations of cortical development subtypes, which have been collectively referred to as “mTORopathies.” Mutations in mTOR regulatory genes such as AKT3 have been associated with epilepsy [37, 38]. Upstream and downstream molecules involved in serine/threonine kinase AKT pathways, including *Akt3,* were found to be involved in epilepsy pathophysiology [32]. Indeed, patients with the risk genotype presented with a high rate of epileptic seizures, but we did not observe a higher seizure rate in our animal model. The animals however, were checked every 4 h (24 h after inoculation) and we did not perform a continuous (video) surveillance for seizures. We previously described that community acquired bacterial meningitis is complicated by seizures in 17 % of episodes and seizures are associated with a high mortality rate (41 %) [6]. Seizures have been associated with severe CNS inflammation and structural CNS lesions, but it is unclear whether the seizures are due to, or cause CNS damage [6].

The genetic variant rs10157763 in AKT3 has been previously associated with aggressive prostate carcinoma and renal cell carcinoma [39]. rs10157763 variant has been described to alter the transcriptional factor binding sites (TFBS) for transcription factors that regulate AKT3 expression.

Genetic variants in *DCTN4* and *RAET1E* were also associated with functional outcome in pneumococcal meningitis, but these findings will need to be validated. DCTN4 is a component of the dynein-dependent motor that moves autophagosomes along microtubules into lysosomes for degradation as part of the autophagy process - a highly conserved cellular quality control mechanism to transport and degrade damaged proteins and microbes [40, 41]. Exome sequencing of extreme phenotypes previously has identified DCTN4 as a modifier of chronic *Pseudomonas aeruginosa* infection in patients with cystic fibrosis [42]. RAET1E is a major histocompatibility complex class 1-like molecule and is a functional ligand for an activating receptor expressed on the surface of immune antigen-presenting cells such as natural killer (NK) cells, CD8(+) T cells, and subsets of CD4(+) T cells [43]. High levels of RAET1E expression on antigen-presenting cells induces down-regulation of NK cell activation by a regulatory T-cell subset [44]. NK cells have been shown to be important for the protection against various microbial pathogens, including *S. pneumoniae* [45], and have shown to contribute to mortality in experimental pneumococcal pneumonia [46]. We did not further study these genes in our animal model as we did not find the genes differentially expressed during meningitis, although this does not completely negate a role in the pathophysiology of pneumococcal meningitis.

Our study has several limitations. First, captured variations with the Human Exome BeadChip are mainly restricted to protein coding regions and used as stand-alone array for identification of the causal variant can be difficult.

Second, some selection bias was possibly introduced since DNA was not available for a considerable proportion of patients (34 %), particularly those with more severe disease. Inclusion of patients with less severe disease will decrease study power to detect genetic variants associated with outcome, resulting in more type II errors (accepting the null hypothesis when it is false). The nationwide design allowed us to detect this selection bias. Third, we could not validate our findings of the genetic association study in a different cohort. The candidate SNPs were associated with unfavourable outcome with a *p*-value lower than 1 × 10^−4^, indicating that findings could still be a type I error. However, no cohorts are currently available for outcome in adult pneumococcal meningitis to validate our results. Therefore, we validated one genetic variant that was upregulated during meningitis in our adult mouse pneumococcal meningitis model [23]. Fourth, to further contribute to elucidate the underlying mechanism(s) on meningitis-associated brain pathology, information regarding tissue protein expression of Akt3 and TNF-α, phenotypical characterization of the cell infiltrate and experiments using TNF- α blocking agents should be assessed in further experiments.

## Conclusions

In conclusion, we identified gene variants in *AKT3, DCTN4* and *RAET1E* to be associated with unfavourable outcome in adults with pneumococcal meningitis. We validated the role of Akt3 in our experimental pneumococcal meningitis model. Our study show that a GWAS approach in pneumococcal meningitis provides new insights in the pathophysiology of pneumococcal disease and may provide new leads for the development of adjunctive therapies.

### Ethics approval and consent to participate

Informed consent was obtained from all individual participants included in the study. The protocol used in this study was approved by the Academic Medical Center, Amsterdam and all local participating hospitals.

All applicable international, national, and/or institutional guidelines for the care and use of animals were followed. All procedures performed in studies involving animals were in accordance with the ethical standards of the institution or practice at which the studies were conducted.

### Consent for publication

Not applicable.

## Additional file

Additional file 1: Table S1. Histopathological scoring system of pneumococcal meningitis. (PDF 12 kb)

## References

[1] Brouwer MC, Tunkel AR, van de Beek D (2010). Epidemiology, diagnosis, and antimicrobial treatment of acute bacterial meningitis. Clin Microbiol Rev.

[2] van de Beek D, de Gans J, Spanjaard L, Weisfelt M, Reitsma JB, Vermeulen M (2004). Clinical features and prognostic factors in adults with bacterial meningitis. N Engl J Med.

[3] Weisfelt M, de Gans J, van der Poll T, van de Beek D (2006). Pneumococcal meningitis in adults: new approaches to management and prevention. Lancet Neurol.

[4] van de Beek D, Schmand B, de Gans J, Weisfelt M, Vaessen H, Dankert J, Vermeulen M (2002). Cognitive impairment in adults with good recovery after bacterial meningitis. J Infect Dis.

[5] Schut ES, Lucas MJ, Brouwer MC, Vergouwen MD, van der Ende A, van de Beek D (2012). Cerebral infarction in adults with bacterial meningitis. Neurocrit Care.

[6] Zoons E, Weisfelt M, de Gans J, Spanjaard L, Koelman JH, Reitsma JB, van de Beek D (2008). Seizures in adults with bacterial meningitis. Neurology.

[7] Hoogman M, van de Beek D, Weisfelt M, de Gans J, Schmand B (2007). Cognitive outcome in adults after bacterial meningitis. J Neurol Neurosurg Psychiatry.

[8] de Gans J, van de Beek D (2002). Dexamethasone in adults with bacterial meningitis. N Engl J Med.

[9] Bijlsma MW, Brouwer MC, Kasanmoentalib ES, Kloek AT, Lucas MJ, Tanck MW, van der Ende A, van de Beek D (2016). Community-acquired bacterial meningitis in adults in the Netherlands, 2006-14: a prospective cohort study. Lancet Infect Dis.

[10] Schut ES, Brouwer MC, de Gans J, Florquin S, Troost D, van de Beek D (2009). Delayed cerebral thrombosis after initial good recovery from pneumococcal meningitis. Neurology.

[11] Brouwer MC, Heckenberg SG, de Gans J, Spanjaard L, Reitsma JB, van de Beek D (2010). Nationwide implementation of adjunctive dexamethasone therapy for pneumococcal meningitis. Neurology.

[12] van de Beek D, de Gans J, Tunkel AR, Wijdicks EF (2006). Community-acquired bacterial meningitis in adults. N Engl J Med.

[13] van de Beek D, Brouwer MC, Thwaites GE, Tunkel AR (2012). Advances in treatment of bacterial meningitis. Lancet.

[14] Woehrl B, Brouwer MC, Murr C, Heckenberg SG, Baas F, Pfister HW, Zwinderman AH, Morgan BP, Barnum SR, van der Ende A, Koedel U, van de Beek D (2011). Complement component 5 contributes to poor disease outcome in humans and mice with pneumococcal meningitis. J Clin Invest.

[15] Brouwer MC, de Gans J, Heckenberg SG, Zwinderman AH, van der Poll T, van de Beek D (2009). Host genetic susceptibility to pneumococcal and meningococcal disease: a systematic review and meta-analysis. Lancet Infect Dis.

[16] Kasanmoentalib ES, Brouwer MC, van de Beek D (2013). Update on bacterial meningitis: epidemiology, trials and genetic association studies. Curr Opin Neurol.

[17] Brouwer MC, Read RC, van de Beek D (2010). Host genetics and outcome in meningococcal disease: a systematic review and meta-analysis. Lancet Infect Dis.

[18] van de Beek D (2012). Progress and challenges in bacterial meningitis. Lancet.

[19] Jennett B, Teasdale G, Braakman R, Minderhoud J, Knill-Jones R (1976). Predicting outcome in individual patients after severe head injury. Lancet.

[20] Guo Y, He J, Zhao S, Wu H, Zhong X, Sheng Q, Samuels DC, Shyr Y, Long J (2014). Illumina human exome genotyping array clustering and quality control. Nat Protoc.

[21] Purcell S, Neale B, Todd-Brown K, Thomas L, Ferreira MA, Bender D, Maller J, Sklar P, de Bakker PI, Daly MJ, Sham PC (2007). PLINK: a tool set for whole-genome association and population-based linkage analyses. Am J Hum Genet.

[22] Easton RM, Cho H, Roovers K, Shineman DW, Mizrahi M, Forman MS, Lee VM, Szabolcs M, de Jong R, Oltersdorf T, Ludwig T, Efstratiadis A, Birnbaum MJ (2005). Role for Akt3/protein kinase Bgamma in attainment of normal brain size. Mol Cell Biol.

[23] Mook-Kanamori B, Geldhoff M, Troost D, van der Poll T, van de Beek D (2012). Characterization of a pneumococcal meningitis mouse model. BMC Infect Dis.

[24] Geldhoff M, Mook-Kanamori BB, Brouwer MC, Troost D, Leemans JC, Flavell RA, Van der Ende A, Van der Poll T, Van de Beek D (2013). Inflammasome activation mediates inflammation and outcome in humans and mice with pneumococcal meningitis. BMC Infect Dis.

[25] Valls Seron M, Duitman J, Geldhoff M, Engelen-Lee J, Havik SR, Brouwer MC, van de Beek D, Spek CA (2015). CCAAT/enhancer-binding protein delta (C/EBPdelta) aggravates inflammation and bacterial dissemination during pneumococcal meningitis. J Neuroinflammation.

[26] Arsenijevic T, Gregoire F, Delforge V, Delporte C, Perret J (2012). Murine 3T3-L1 adipocyte cell differentiation model: validated reference genes for qPCR gene expression analysis. PLoS One.

[27] Chang CC, Chow CC, Tellier LC, Vattikuti S, Purcell SM, Lee JJ (2015). Second-generation PLINK: rising to the challenge of larger and richer datasets. Gigascience.

[28] Adzhubei IA, Schmidt S, Peshkin L, Ramensky VE, Gerasimova A, Bork P, Kondrashov AS, Sunyaev SR (2010). A method and server for predicting damaging missense mutations. Nat Methods.

[29] Weisfelt M, van de Beek D, Spanjaard L, Reitsma JB, de Gans J (2006). Clinical features, complications, and outcome in adults with pneumococcal meningitis: a prospective case series. Lancet Neurol.

[30] Mook-Kanamori BB, Geldhoff M, van der Poll T, van de Beek D (2011). Pathogenesis and pathophysiology of pneumococcal meningitis. Clin Microbiol Rev.

[31] Engelen-Lee JY, Brouwer MC, Aronica E, van de Beek D (2016). Pneumococcal meningitis: Clinical-pathological correlations (meningene-path). Acta Neuropathol Commun.

[32] Tokuda S, Mahaffey CL, Monks B, Faulkner CR, Birnbaum MJ, Danzer SC, Frankel WN (2011). A novel Akt3 mutation associated with enhanced kinase activity and seizure susceptibility in mice. Hum Mol Genet.

[33] Dudek H, Datta SR, Franke TF, Birnbaum MJ, Yao R, Cooper GM, Segal RA, Kaplan DR, Greenberg ME (1997). Regulation of neuronal survival by the serine-threonine protein kinase Akt. Science.

[34] Datta SR, Brunet A, Greenberg ME (1999). Cellular survival: a play in three Akts. Genes Dev.

[35] Brinkmann V, Reichard U, Goosmann C, Fauler B, Uhlemann Y, Weiss DS, Weinrauch Y, Zychlinsky A (2004). Neutrophil extracellular traps kill bacteria. Science.

[36] Tsiperson V, Gruber RC, Goldberg MF, Jordan A, Weinger JG, Macian F, Shafit-Zagardo B (2013). Suppression of inflammatory responses during myelin oligodendrocyte glycoprotein-induced experimental autoimmune encephalomyelitis is regulated by AKT3 signaling. J Immunol.

[37] Crino PB. mTOR signaling in epilepsy: insights from malformations of cortical development. Cold Spring Harb Perspect Med. 2015;5(4). doi:10.1101/cshperspect.a022442. 10.1101/cshperspect.a022442 PMC4382721 25833943

[38] Cohen MM (2013). The AKT genes and their roles in various disorders. Am J Med Genet A.

[39] Lavender NA, Rogers EN, Yeyeodu S, Rudd J, Hu T, Zhang J, Brock GN, Kimbro KS, Moore JH, Hein DW, Kidd LC (2012). Interaction among apoptosis-associated sequence variants and joint effects on aggressive prostate cancer. BMC Med Genomics.

[40] Kimura S, Noda T, Yoshimori T (2008). Dynein-dependent movement of autophagosomes mediates efficient encounters with lysosomes. Cell Struct Funct.

[41] Haspel JA, Choi AM (2011). Autophagy: a core cellular process with emerging links to pulmonary disease. Am J Respir Crit Care Med.

[42] Emond MJ, Louie T, Emerson J, Zhao W, Mathias RA, Knowles MR, Wright FA, Rieder MJ, Tabor HK, Nickerson DA, Barnes KC, Gibson RL, Bamshad MJ (2012). Exome sequencing of extreme phenotypes identifies DCTN4 as a modifier of chronic Pseudomonas aeruginosa infection in cystic fibrosis. Nat Genet.

[43] Ogasawara K, Lanier LL (2005). NKG2D in NK and T cell-mediated immunity. J Clin Immunol.

[44] Lin Z, Wang C, Xia H, Liu W, Xiao W, Qian L, Jia X, Ding Y, Ji M, Gong W (2014). CD4(+) NKG2D(+) T cells induce NKG2D down-regulation in natural killer cells in CD86-RAE-1epsilon transgenic mice. Immunology.

[45] Zajonc DM, Girardi E (2015). Recognition of microbial glycolipids by natural killer T cells. Front Immunol.

[46] Christaki E, Diza E, Giamarellos-Bourboulis EJ, Papadopoulou N, Pistiki A, Droggiti DI, Georgitsi M, Machova A, Lambrelli D, Malisiovas N, Nikolaidis P, Opal SM (2015). NK and NKT cell depletion alters the outcome of experimental pneumococcal pneumonia: relationship with regulation of interferon-gamma production. J Immunol Res.

