# Supplementary material for: V-akt murine thymoma viral oncogene homolog 3 (AKT3) contributes to poor disease outcome in humans and mice with pneumococcal meningitis
Source: Acta Neuropathol Commun. 2016 May 18;4:50. doi: 10.1186/s40478-016-0320-9 (PMC4870776; doi:10.1186/s40478-016-0320-9)
Supplement: Additional file 1: Table S1. — Histopathological scoring system of pneumococcal meningitis. (PDF 12 kb) [file 40478_2016_320_MOESM1_ESM.pdf]

ONLINE RESOURCE TABLE

Table 1. Histopathological scoring system of pneumococcal meningitis.

**Scoring criteria**		**Scores**			
**Main category**	**Subcategory**	**0**	**1**	**2**	**3**
Meningeal infiltration		Absent	Focal mild infiltration	Multifocal mild or focal severe infiltration	Multifocal severe infiltration
Parenchymal damage	Parenchymal infiltration of inflammatory cells	Absent	Focal mild infiltration	Multifocal mild or focal severe infiltration	Multifocal severe infiltration
	Infarction<br>Haemorrhage<br>Abscess	Absent	Focal small damage	Multifocal small or focal large damage	Multifocal large damages
Vascular inflammation	Large meningeal artery inflammation<br>Small parenchymal vessel inflammation	Absent	Focal mild (sub)endothelial infiltration /reactive changes	Multifocal mild (sub)endothelial infiltration /reactive changes or focal severe (sub)-endothelial infiltration with obstruction of vascular lumen and/or extension of infiltration in the media layer	Multifocal severe (sub)endothelial infiltration with obstruction of vascular lumen and/or extension of infiltration in the media layer
Thrombosis	Arterial thrombosis<br>Venous thrombosis<br>Small vessel thrombosis	Absent	Focal mild with partial obstruction of vascular lumen	Multifocal mild with partial obstruction of vascular lumen or focal severe with complete obstruction of vascular lumen and destruction of vessel wall	Multifocal severe with complete obstruction of vascular lumen and destruction of vessel wall
Ventriculitis		Absent	A few inflammatory cells in the ventricle	Groups of inflammatory cells in the ventricle with/without ependymal infiltration	Extension of inflammatory cells into the periventricular tissue
